# Platelet-to-lymphocyte ratio as a predictor of preoperative venous thrombosis in femoral fracture patients: a retrospective cohort study

**DOI:** 10.3389/fcvm.2025.1672545

**Published:** 2026-01-20

**Authors:** Kunlun Li, Delun Li, Jianguang Sun

**Affiliations:** 1 Department of Traumatic Orthopedics, Meizhou People’s Hospital, Meizhou, China; 2 Data Center, Meizhou People’s Hospital, Meizhou, China

**Keywords:** femoral fracture, vein thrombosis, lower extremity, inflammation and immune indexes platelet-to-lymphocyte ratio, neutrophil-to-lymphocyte ratio

## Abstract

**Objective:**

The aim of this study was to investigate the relationship between the platelet-to-lymphocyte ratio (PLR) and the risk of lower extremity deep vein thrombosis (DVT) in patients with femoral fracture, and to evaluate the potential influence of other risk factors, including age, gender, smoking status, neutrophil-to-lymphocyte ratio (NLR), and multiple fractures of the lower extremities.

**Methods:**

A retrospective study was conducted on 1,083 patients with femoral fractures treated at Meizhou People's Hospital between November 2017 and April 2024. DVT was diagnosed using Doppler ultrasound. Data on clinical features, including age, gender, body mass index, history of smoking, hypertension, diabetes mellitus, and multiple fractures of the lower extremities, were collected. Routine blood tests were performed at admission to calculate inflammatory indices, including PLR, NLR, and others. Logistic regression analysis was used to assess the independent association of these factors with DVT.

**Results:**

Among the 1,083 patients, 218 (20.1%) developed DVT. Logistic regression analysis identified that PLR (OR = 1.74, 95% CI: 1.16–2.62, *P* = 0.008), age (OR = 1.9, 95% CI: 1.21–3.30, *P* = 0.007), NLR (OR = 2.08, 95% CI:1.24–3.48, *P* = 0.005), gender (OR = 1.70, 95% CI: 1.17–2.49=, *P* = 0.005), history of smoking (OR = 2.19, 95% CI: 1.00–4.77, *P* = 0.05), and multiple fractures of the lower extremities (OR = 2.02, 95% CI: 1.32–3.11, *P* = 0.001) were independent risk factors for DVT.

**Conclusions:**

PLR is an independent risk factor for lower extremity DVT in patients with femoral fracture, with a modest predictive performance (AUC = 0.60) that slightly outperforms age and NLR in this cohort. While these findings suggest PLR may have potential as a supplementary biomarker for DVT risk stratification, its clinical utility requires further validation in larger, multicenter studies.

## Introduction

Deep vein thrombosis (DVT) is a significant global health concern, with an annual incidence of 1–2 cases per 1,000 individuals and a high risk of life-threatening complications such as pulmonary embolism (1, 2). Despite advances in prophylaxis, DVT remains prevalent in high-risk populations, particularly patients with femoral fractures, where prolonged immobilization and systemic inflammation exacerbate thrombotic risk (3, 4). The pathogenesis of DVT is driven by Virchow's triad—venous stasis, hypercoagulability, and endothelial injury (5). Traditional risk factors include age, obesity, and diabetes (6, 7). Recent research has highlighted the potential of immune-inflammatory markers, including the platelet-to-lymphocyte ratio (PLR), neutrophil-to-lymphocyte ratio (NLR), and systemic immune-inflammation index (SII), as predictors of thrombotic risk (8, 9).

Among these biomarkers, PLR has emerged as particularly promising due to its unique ability to reflect both thrombotic (platelets) and inflammatory (lymphocytes) pathways (10, 11). Supporting this rationale, Çiçek et al. (12) recently demonstrated the diagnostic value of platelet-based ratios in acute limb embolism, reinforcing the biological plausibility of such indices in thromboembolic conditions. While NLR and SII have been extensively studied in other contexts (13, 14), PLR offers distinct advantages for DVT prediction in femoral fracture patients, including superior clinical performance (higher sensitivity and specificity) in preliminary studies (15, 16) and practical utility as it can be derived from routine complete blood counts (17).

This study specifically evaluates PLR as a predictor of preoperative DVT in femoral fracture patients, addressing a critical gap in the current literature (18, 19). By validating PLR's predictive value in this high-risk population, we aim to provide clinicians with a reliable, cost-effective tool for early DVT risk assessment. The findings could significantly impact clinical practice by improving risk stratification and guiding prophylactic strategies for this vulnerable patient group (20, 21).

## Methods

### Patients

**Figure 1:**
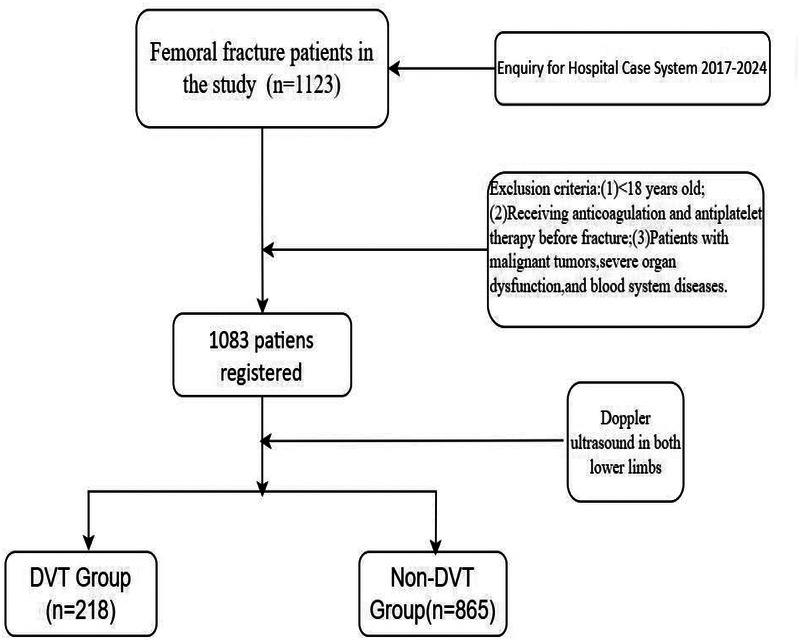
The workflow of patients in our study. DVT, deep vein thrombosis.

A total of 1,083 patients with femoral fractures receiving treatment at Meizhou People's Hospital were selected between November 2017 and April 2024. Vein thrombosis was diagnosed by Doppler ultrasound of both lower limbs. This standardized approach ensured universal screening regardless of clinical suspicion, minimizing selection bias. Ultrasound examinations were performed by trained vascular specialists within 48 h of admission, following a predefined institutional protocol mandating Doppler screening for all traumatic lower limb fracture patients. This protocol aligns with recent guidelines emphasizing early DVT detection in high-risk orthopedic populations (17). Inclusion criteria were as follows: (1) fresh femur fracture; (2) patients with fractures of all parts of femur; (3) patients ≥18 years; and (4) no pre-existing limb, motor, and sensory dysfunction prior to fracture. Exclusion criteria were as follows: (1) patients <18 years; (2) patients receiving anticoagulation and antiplatelet therapy prior to fracture; and (3) patients with malignant tumors, severe organ dysfunction, or blood system diseases. This study was supported by the Ethics Committee of the Meizhou People's Hospital (2022-C-116) (Figure 1).

### Data collection

Clinical features of the patients were collected from the hospital’s medical records system, including gender, age, body mass index (BMI), history of smoking, history of alcohol consumption, hypertension, diabetes mellitus, multiple fractures of lower extremities, and lower extremity vein thrombosis. Routine blood test data were collected at admission. A routine blood routine test was also conducted, which involved collecting 2 mL of the patient's blood sample in a test tube through via venipuncture of an antecubital vein. Ethylenediaminetetraacetic acid (EDTA) was used as an anticoagulant and the sample was tested using a Sysmex XE-2100 hematology analyzer (Sysmex Corporation, Japan) according to standard operating procedures (SOP).

In this study, we employed a comprehensive approach to evaluate the relationship between inflammatory and immune markers and the risk of lower extremity vein thrombosis in patients with femoral fractures. The calculation of inflammation indices was based on previously validated methods (13, 14). All blood parameters (platelet, neutrophil, lymphocyte, and monocyte counts) were measured in ×10⁹/L, as per standardized clinical laboratory protocols. In particular, we calculated the following indices using routine blood test parameters:

Systemic Immune-Inflammatory Index (SII):

SII = platelet count (×10⁹/L)×neutrophil count (×10⁹/L)/lymphocyte count (×10⁹/L)SII = platelet count (×10⁹/L) × neutrophil count (×10⁹/L)/lymphocyte count (×10⁹/L)

Systemic Inflammatory Response Index (SIRI):

SIRI = monocyte count (×10⁹/L)×neutrophil count (×10⁹/L)/lymphocyte count (×10⁹/L)SIRI = monocyte count (×10⁹/L)×neutrophil count (×10⁹/L)/lymphocyte count (×10⁹/L)

Neutrophil-to-Lymphocyte Ratio (NLR):

NLR = neutrophil count (×10⁹/L)/lymphocyte count (×10⁹/L)NLR = neutrophil count (×10⁹/L)/lymphocyte count (×10⁹/L)

Platelet-to-Lymphocyte Ratio (PLR):

PLR = platelet count (×10⁹/L)/lymphocyte count (×10⁹/L)PLR = platelet count (×10⁹/L)/lymphocyte count (×10⁹/L)

Monocyte-to-Lymphocyte Ratio (MLR):

MLR = monocyte count (×10⁹/L)/lymphocyte count (×10⁹/L)MLR = monocyte count (×10⁹/L)/lymphocyte count (×10⁹/L)

Systemic Immune-Inflammatory Index (SII): This index is a composite marker reflecting the interaction between platelets and leukocytes. It is calculated as SII = platelet × neutrophil/lymphocyte. The SII has been widely used to assess the inflammatory status in various diseases, including cancer and cardiovascular disorders (13).

Systemic Inflammatory Response Index (SIRI): This index incorporates monocytes, neutrophils, and lymphocytes to reflect the overall inflammatory response. It is calculated as SIRI = monocyte × neutrophil/lymphocyte. SIRI has been shown to be a reliable marker for predicting disease severity and prognosis (13).

Neutrophil-to-Lymphocyte Ratio (NLR): This ratio reflects the balance between pro-inflammatory neutrophils and anti-inflammatory lymphocytes. It is calculated as

NLR = neutrophil/lymphocyte. NLR has been extensively studied in various inflammatory and thrombotic conditions (15).

Platelet-to-Lymphocyte Ratio (PLR): This ratio indicates the interaction between platelets and lymphocytes, which is crucial for understanding the thrombotic risk. It is calculated as PLR = platelet/lymphocyte. Elevated PLR has been associated with increased thrombotic events in several studies (10).

Monocyte-to-Lymphocyte Ratio (MLR): This ratio reflects the relative abundance of monocytes and lymphocytes, which may influence the inflammatory cascade. It is calculated as MLR = monocyte/lymphocyte. MLR has been reported to be a potential marker for inflammation and thrombosis (16).

### Statistical analysis

Statistical analysis was performed using SPSS version 26.0 (IBM Corp., USA). Categorical variables were compared using Chi-square test or Fisher's exact test. Logistic regression analysis was employed to evaluate the independent association between inflammatory markers and the risk of vein thrombosis, with statistical significance defined as *P* < 0.05.

## Results

### Clinical features of patients with femoral fracture

Among the 1,083 patients with femoral fracture included in this study, 516 (47.6%) were male and 567 (52.4%) were female. The age distribution was as follows: 112 (10.3%) were <45 years, 166 (15.3%) were 45–59 years, and 805 (74.3%) were >59 years. Regarding BMI, 154 patients (14.8%) had a BMI <18.5 kg/m^2^, 560 (53.6%) had a BMI of 18.5–23.9 kg/m^2^, and 330 (31.6%) had a BMI ≥24.0 kg/m^2^. In addition, 62 patients (5.7%) had a history of smoking, 14 (1.3%) had a history of alcohol consumption, 294 (27.1%) had hypertension, and 162 (15.0%) had diabetes mellitus. Multiple fractures of the lower extremities were present in 215 (19.9%) patients, and 218 (20.1%) patients had vein thrombosis of the lower extremity. The mean levels of SII, SIRI, NLR, PLR, and MLR were 1,153.3 (IQR 774.8–1,772.8), 3.9 (IQR 2.2–6.6), 5.8 (IQR 3.9–8.9), 186.2 ± 111.8, and 0.5 (IQR 0.4–0.8), respectively (Table 1).

**Table 1:** Clinical and demographic characteristics of femoral fracture patients with and without DVT.

Variables	Total (*n* = 1,083)	Non-DVT (*n* = 865)	DVT (*n* = 218)	*p*	Statistic
Gender, *n* (%)				0.035	4.4
Male, *n* (%)	516 (47.6)	426 (49.2)	90 (41.3)		
Female, *n* (%)	567 (52.4)	439 (50.8)	128 (58.7)		
BMI, *n* (%)				0.138	4.0
<18.5, *n* (%)	154 (14.8)	123 (14.2)	31 (17.3)		
18.5–23.9, *n* (%)	560 (53.6)	476 (55)	84 (46.9)		
≥24.0, *n* (%)	330 (31.6)	266 (30.8)	64 (35.8)		
Age, *n* (%)				<0.001	15.6
<45, *n* (%)	112 (10.3)	100 (11.6)	12 (5.5)		
45–59	153 (14.1)	134 (15.5)	19 (8.7)		
>59, *n* (%)	818 (75.5)	631 (72.9)	187 (85.8)		
MLR, median (IQR)	0.5 (0.4, 0.8)	0.5 (0.4, 0.7)	0.6 (0.4, 0.9)	<0.001	11.5
PLR, mean ± SD	186.2 ± 111.8	175.2 ± 86.7	229.9 ± 173.3	<0.001	43.3
NLR, median (IQR)	5.8 (3.9, 8.9)	5.7 (3.7, 8.4)	6.5 (4.5, 11.1)	<0.001	18.6
SIRI, median (IQR)	3.9 (2.2, 6.6)	3.7 (2.2, 6.4)	4.3 (2.4, 7.8)	0.005	8.0
SII, median (IQR)	1,153.3 (774.8, 1,772.8)	1,114.2 (748.3, 1,658.7)	1,347.7 (855.6, 2,343.1)	<0.001	17.3
Multiple fractures of lower extremities				0.142	2.2
No, *n* (%)	868 (80.1)	701 (81)	167 (76.6)		
Yes, *n* (%)	215 (19.9)	164 (19)	51 (23.4)		
History of alcohol consumption				0.498	Fisher
No, *n* (%)	1,069 (98.7)	855 (98.8)	214 (98.2)		
Yes, *n* (%)	14 (1.3)	10 (1.2)	4 (1.8)		
History of smoking				0.251	1.3
No, *n* (%)	1,021 (94.3)	819 (94.7)	202 (92.7)		
Yes, *n* (%)	62 (5.7)	46 (5.3)	16 (7.3)		
Diabetes mellitus				0.611	0.3
No, *n* (%)	921 (85.0)	738 (85.3)	183 (83.9)		
Yes, *n* (%)	162 (15.0)	127 (14.7)	35 (16.1)		
Hypertension				0.002	9.2
No, *n* (%)	789 (72.9)	648 (74.9)	141 (64.7)		
Yes, *n* (%)	294 (27.1)	217 (25.1)	77 (35.3)		

BMI, body mass index; SII, systemic immune-inflammatory index; SIRI, systemic inflammatory response index; NLR, neutrophil-to-lymphocyte ratio; PLR, platelet-to-lymphocyte ratio; MLR, monocyte-to-lymphocyte ratio. IQR, interquartile range; SD, standard deviation.

Logistic regression analysis was performed to measure the relationship between the associated factors and vein thrombosis of the lower extremity. The results of univariate analysis showed that gender, age, hypertension, SII, SIRI l, NLR, PLR, and MLR were significantly associated with vein thrombosis of the lower extremity. Multivariate regression logistic analysis identified that gender (OR =1.71,95% CI:1.171–2.49, *P* = 0.005), age (OR = 2.0,95% CI:1.21–3.30, *P* = 0.007), history of smoking (OR = 2.19, 95% CI:1.01–4.77, *P* = 0.048), multiple fractures of lower extremities (OR = 2.02,95% CI: 1.32–3.11, *P* = 0.001), NLR (OR = 2.08, 95% CI:1.24–3.48,*P* = 0.005), and PLR (OR = 1.74, 95% CI:1.16–2.62,*P* = 0.008) were independent risk factors for vein thrombosis of the lower extremity after femoral fracture (Table 2).

**Table 2:** Logistic regression analysis of risk factors for lower extremity DVT.

Variables	Univariate		Multivariate	
	OR (95% CI)	*p* values	OR (95% CI)	*p* values
Gender (female/male）	1.380 (1.022–1.864)	0.036	1.706 (1.171–2.486)	0.005
Age (≥60/<60, years old)	2.126 (1.433–3.155)	<0.001	1.997 (1.209–3.299)	0.007
BMI (kg/m^2^)				
18.5–23.9	1.000 (reference)	–	1.000 (reference)	–
<18.5	1.372 (0.866–2.176)	0.178	1.213 (0.750–1.961)	0.432
≥24.0	1.337 (0.935–1.913)	0.112	1.456 (0.989–2.142)	0.057
History of smoking (yes/no)	1.410 (0.782–2.542)	0.253	2.191 (1.006–4.774)	0.048
History of alcoholism (yes/no)	1.598 (0.496–5.145)	0.432	1.061 (0.230–4.893)	0.940
Hypertension (yes/no)	1.631 (1.187–2.240)	0.003	1.462 (0.996–2.145)	0.052
Diabetes mellitus (yes/no)	1.111 (0.739–1.671)	0.612	1.073 (0.671–1.716)	0.768
Multiple fractures of lower extremities (yes/no)	1.305 (0.914–1.865)	0.143	2.022 (1.316–3.107)	0.001
SII (≥1,294.2/<1,294.2)	1.797 (1.333–2.424)	<0.001	0.870 (0.553–1.368)	0.546
SIRI (≥3.655/<3.655)	1.571 (1.159–2.130)	0.004	0.774 (0.468–1.278)	0.317
NLR (≥3.995/<3.995)	2.305 (1.547–3.432)	<0.001	2.082 (1.244–3.485)	0.005
PLR (≥197.95/<197.95)	2.063 (1.526–2.791)	<0.001	1.741 (1.157–2.621)	0.008
MLR (≥0.695/<0.695)	1.880 (1.383–2.555)	<0.001	1.513 (0.948–2.415)	0.082

BMI, body mass index; SII, systemic immune-inflammatory index; SIRI, systemic inflammatory response index; NLR, neutrophil-to-lymphocyte ratio; PLR, platelet-to-lymphocyte ratio; MLR, monocyte-to-lymphocyte ratio; CI, confidence interval; OR, odds ratio.

ROC analysis indicated that PLR (AUC=0.60) and NLR (AUC=0.59) had comparable predictive performance (*P* = 0.714), with PLR offering higher sensitivity (69%) and NLR higher specificity (85%) at their optimal cutoffs.

The predictive performance of age, NLR, and PLR for lower extremity DVT was evaluated using ROC curve analysis, with results detailed in Table 3. The AUC values were 0.59 (95% CI: 0.56–0.62) for age, 0.58 (95% CI: 0.55–0.64) for NLR, and 0.60 (95% CI: 0.56–0.65) for PLR. Optimal cutoff values were identified as 79.5 years for age, 3.99 for NLR, and 198 for PLR, corresponding to sensitivity/specificity of 67%/48% for age, 29%/85% for NLR, and 69%/49% for PLR. All variables demonstrated statistically significant *P*-values <0.001.

**Table 3:** The predictive ability of Age, NLR, and PLR for lower extremity deep vein thrombosis (DVT).

Variables	AUC	95% CI	Sensitivity (%)	Specificity (%)	Cutoff	*P*
Age	0.58	0.60–0.66	0.67	0.48	79.5	<0.001
NLR	0.59	0.55–0.64	0.29	0.85	4.0	<0.001
PLR	0.60	0.56–0.65	0.69	0.49	198.0	<0.001

NLR, neutrophil-to-lymphocyte ratio; PLR, platelet-to-lymphocyte ratio.

## Discussion

DVT, a disorder of venous return in the lower extremities, is a common complication in patients with lower extremity fractures after surgery. Pathologically, blood abnormally agglutinates and blocks vein lumen in deep veins under conditions of vascular wall injury and blood hypercoagulability. Clinically, diagnosis of vein thrombosis is challenging, as patients often present with local pain and edema, leading to the condition often being overlooked. Notably, thrombus shedding can lead to pulmonary embolism, a severe life-threatening development (22).

In line with previous studies, in this study, gender, age, history of smoking, and multiple fractures of lower extremities were independent risk factors for vein thrombosis of lower extremities in patients with femoral fractures. Consistent with our findings, Zhang et al. found that age >50 years, female sex, and cigarette smoking were independent risk factors for vein thrombosis in patients with traumatic fractures (18). Extending the evidence base, there have been several studies reported on risk factors for lower extremity vein thrombosis in patients with lower extremity fractures. Similarly, Chang et al. revealed that advanced age and diabetes mellitus were independent risk factors for lower extremity fractures complicated by deep vein thrombosis (19). Contrastingly, Zuo et al. found that obesity (BMI ≥ 24.0 kg/m^2^) was an independent factor for vein thrombosis of lower extremities after intertrochanteric fracture (15). Interestingly, male sex was a risk factor for vein thrombosis in ankle fractures (20) and tibial plateau fractures (11). Conversely, female sex was a risk factor for vein thrombosis in patients with traumatic fractures (18). In addition, obesity was associated with vein thrombosis in foot fractures (14). Supporting this, Chang et al. found that diabetes mellitus was a potential risk factor for vein thrombosis in patients with lower extremity fractures (19). Notably, diabetes mellitus was an independent risk factor for vein thrombosis in patients with femoral neck fractures (13). Collectively, some studies found that advanced age was found to be a risk factor for vein thrombosis in patients with lower extremity fractures (12, 19, 23–25). In particular, age ≥65 years was identified as a risk factor of vein thrombosis in closed patella fractures (26). Similarly, age ≥65 years was identified as a risk factor of vein thrombosis in lower extremities after hip fractures (27). Moreover, age ≥40 years was a risk factor of vein thrombosis in patients with tibial fractures (28). Importantly, patients with multiple injuries have a higher risk of deep vein thrombosis (29, 30). The results of those studies are consistent with the findings of the present study. Furthermore, Ma et al. found that alcohol consumption was associated with vein thrombosis in foot fractures (14). Hypertension was a risk factor for vein thrombosis of lower extremities in tibial plateau fractures. However, this study did not reach similar conclusions.

Mechanistically, in this study, high PLR (≥198), and high NLR (≥4.0) were identified as independent risk factors for vein thrombosis of lower extremities in patients with femoral fractures. Contextually, there are relatively few studies analyzing the relationship between inflammation and immune indexes and venous thrombosis after lower limb fractures. Fundamentally, neutrophil extracellular traps released by neutrophils are involved in the formation of vein thrombosis in patients with traumatic fractures (31). Luo et al. found that reduced lymphocyte count was independently associated with vein thrombosis in patients with ankle fractures (32). Contrastingly, Liu et al. revealed that the occurrence of vein thrombosis after tibial plateau fractures was independently related to the levels of platelets and neutrophils, but not NLR, PLR, MLR, and SII (13). Supporting our findings, Zeng et al. found that high NLR and SII were independent predictors of vein thrombosis among patients with intertrochanteric femoral fractures (33). In addition, high SII was a predictor of vein thrombosis in elderly patients with hip fractures (34).

Beyond cellular markers, Luo et al. identified that lower albumin levels, reduced lymphocyte counts, and elevated D-dimer levels were associated with vein thrombosis of the lower extremities following ankle fractures. Overall, platelet distribution width (PDW), high-density lipoprotein cholesterol (HDL-C), serum alkaline phosphatase (ALP), serum sodium concentration, and D-dimer levels were associated with vein thrombosis following foot fractures (14). Zhang et al. proposed that an innovative fibrinolysis index [calculated by lysis potential (LP), lysis time (LT), blood cell counts, conventional coagulation tests, and tissue plasminogen activator inhibitor complex (tPAIC)] could be a biomarker for predicting vein thrombosis after traumatic lower extremity fractures (35). Consistently, some studies found that there was a significant difference in D-dimer levels between thrombus patients and non-thrombus patients after lower limb fractures (18, 25, 36, 37). Extending these findings, Zhu et al. found that alkaline phosphatase, sodium concentration, and D-dimer were associated with vein thrombosis of the lower extremities following tibial plateau fractures (11). Notably, abnormal lactic dehydrogenase (LDH), low serum sodium concentrations, and higher hematocrit levels were independently associated with vein thrombosis after hip fractures (38).

This study is one of the few to examine the relationship between levels of peripheral blood immune-inflammatory markers and the risk of vein thrombosis of lower extremities following femoral fractures. Methodologically, this study still has the following limitations: (1) This study is a single-center retrospective study, which inevitably has selection bias. The findings need to be confirmed in large, multicenter studies. (2) Given the limitations of vascular ultrasonography, the results of this study do not exclude the possibility of false positives and false negatives in the diagnosis of vein thrombosis of lower extremities. (3) As some patients did not undergo long-term follow-up after discharge, the clinical outcome of preoperative vein thrombosis was not evaluated in this study.

Building on recent advances, Çiçek et al. (12) demonstrated the diagnostic value of red blood cell distribution-to-platelet ratio (RPR) in acute leg embolism, providing a comparative context for our PLR findings. Furthermore, Songur et al. (39) established PLR's prognostic significance in arterial pathologies, suggesting its broader vascular relevance beyond venous thrombosis.

## Conclusions

PLR is an independent risk factor for lower extremity DVT in patients with femoral fractures, with a modest predictive performance (AUC = 0.60) that slightly outperforms age and NLR in this cohort. Although these findings suggest that PLR may have potential as a supplementary biomarker for DVT risk stratification, its clinical utility requires further validation in larger, multicenter studies.

## Data Availability

The original contributions presented in the study are included in the article/Supplementary Material; further inquiries can be directed to the corresponding author.
